# Weight analysis of Chinese nurses' behaviors to maintain patient dignity and its relationship with job-esteem: a cross-sectional study controlling for agreeableness

**DOI:** 10.3389/fpsyg.2025.1710563

**Published:** 2026-01-07

**Authors:** Cong Guo, Chunlin Zhang, Cuizhu Zhou, Mengqi Zhu, Lingling Chen, Youran Liu, Yequn Zhang, Jie Wang, Tengfei Liang

**Affiliations:** 1 School of Nursing, Bengbu Medical University, Anhui, Bengbu, China; 2 The Second Affiliated Hospital of Bengbu Medical University, Anhui, Bengbu, China; 3 The Nethersole School of Nursing, Faculty of Medicine, The Chinese University of Hong Kong, Hong Kong SAR, China

**Keywords:** agreeableness, cross-sectional study, job-esteem, nurses, patient dignity

## Abstract

**Background:**

Patient dignity is fundamental to nursing ethics and care quality, yet nurses often face challenges in upholding it. This study examines how nurses' Job-Esteem influences their behaviors for maintaining patient dignity in China.

**Methods:**

This two-stage study was conducted in 2025. The first round (April) employed convenience sampling at a hospital in Anhui Province, yielding 508 valid responses. The second round (November) utilized a multi-stage random sampling across five hospitals in East and Central China, yielding 496 valid responses, resulting in a total of 1,004 valid questionnaires. All participants were assessed using the Dignity in Care Scale for Nurses, Job-Esteem Scale for Nurses in Hospital, and Chinese Big Five Personality Inventory brief version. Hierarchical regression was used for analysis.

**Results:**

The total score of Nurses' Behaviors to Maintain Patient Dignity was 168.00(145.00,180.00), and the total score of Job-Esteem was 112.00(99.00,124.00). Job-Esteem explained an additional 42% of the variance in Nurses‘ Behaviors to Maintain Patient Dignity. Key predictors included professional competence (β = 0.29), professional self-awareness (β = 0.18), respect and recognition of the organization (β = 0.19), and social trust and Respect (β = 0.15). Weight analysis indicated that the “Patient Care Needs Promptly (PCNP)” dimension had the highest weight (17.50%).

**Conclusion:**

Job-Esteem was significantly associated with Nurses' Behaviors to Maintain Patient Dignity. Our findings suggest that interventions focused on enhancing professional competence, strengthening organizational support, and addressing Patient Care Needs Promptly may be promising avenues for advancing dignity-based care.

## 1 Introduction

The International Council of Nurses (ICN) (Stievano and Tschudin, 2019) emphasizes in its Code of Ethics for Nurses that “dignity, as a reflection of the essence of humanity, encompasses profound meanings such as being heard and understood, respected and trusted.” The Code further highlights that “maintaining patient dignity and respect for personhood constitutes a core ethical principle that healthcare professionals—particularly nurses—must uphold in clinical practice, requiring nurses to actively understand patients‘ cultural backgrounds, values, and individual needs.” As direct providers of healthcare services, nurses play a vital role in preserving patient dignity. Although dignity preservation should be integrated throughout the entire nursing process, its implementation remains challenging in practice due to factors such as cultural diversity and increasing clinical workloads (Cheraghi et al., 2014; Lin et al., 2013). Studies indicate that emergency department patients often experience compromised dignity during care, manifested through insufficient privacy protection and limited autonomy in decision-making (Fuseini et al., 2022). Such issues not only harm patients' physical and mental health but are also significantly negatively correlated with core indicators of humanistic care quality in medical institutions (Lam, 2007). Conversely, effective dignity maintenance can accelerate patient recovery, improve treatment compliance (Ferri et al., 2015; Khademi et al., 2012), and serve as a key indicator in evaluating patient satisfaction (Helali Sotoodeh et al., 2024). Therefore, the ability of nurses to maintain patient dignity is crucial in clinical practice. In the current context of widespread strain on medical resources, relying solely on external regulatory mechanisms is insufficient for sustainably improving the quality of dignity preservation. Thus, there is a practical need to explore the intrinsic motivational factors that drive nurses to uphold patient dignity.

Nurses‘ professional psychological traits—particularly Job-Esteem and personality characteristics such as Agreeableness—profoundly influence the quality of care they provide by shaping clinical decision-making processes and personal sense of responsibility (Fagermoen, 1997). Agreeableness refers to a personality trait characterized by cooperativeness, compassion, and tolerance (McCrae and Costa, 1987), with altruism being one of its components (Hastings and O'Neill, 2009). Extensive research has demonstrated a significant positive correlation between agreeableness and ethical behavior (Drach-Zahavy and Srulovici, 2019; King et al., 2005), indicating that nurses high in agreeableness are more likely to engage in helping behaviors. Job-Esteem of nurses represents another psychological trait, encompassing values and beliefs related to the nursing profession. It includes nurses' professional self-perception, intuition in nursing practice, and professionalism sustained throughout their careers (Choi and Jung, 2020). This sense of Job-Esteem not only influences nurses' job satisfaction but is also an important predictor of clinical care quality (Lee and Kim, 2023). It stems from internal recognition of the profession's value and external feedback. Specifically, through adhering to professional ethics, nurses gradually build professional self-confidence, enabling them to form positive perceptions regarding the social value of nursing and the level of organizational recognition they receive (Choi and Jung, 2020). When nurses consistently receive positive feedback from patients or organizational support, their Job-Esteem enhances ethical sensitivity in clinical decision-making, motivating them to more proactively safeguard core dimensions of patient dignity such as autonomy and privacy (Dehghani et al., 2015). Conversely, a lack of Job-Esteem can initiate a vicious cycle: diminished self-worth leads to burnout, which in turn reduces care quality (Parandeh et al., 2016; Sabatino et al., 2014) and ultimately weakens the protection of patient dignity. Notably, Job-Esteem not only indirectly improves dignity maintenance by enhancing work engagement and emotional support capacity (Jang et al., 2016) but empirical studies also suggest that eliminating factors that undermine nurses' professional dignity is a key pathway to ensuring respectful patient care (Abbasi et al., 2023).

However, critical gaps remain regarding the specific relationship between Job-Esteem and Nurses‘ behaviors for maintaining patient dignity. First, existing studies examining this relationship often fail to adequately control for potential confounding variables. For example, nurses' personality traits have been shown to significantly influence their clinical decision-making styles, communication approaches, and responsiveness to patient needs (Okumura et al., 2022), but since personality traits may themselves be correlated with Job-Esteem, it remains difficult to clearly delineate the independent effect of Job-Esteem on dignity-maintaining behaviors. Second, there is a notable lack of empirical evidence specifically focused on Chinese nurses. Nurses in China face unique professional challenges, including pervasive social biases such as “prioritizing medicine over nursing” and chronically excessive workloads (Shi et al., 2004; Zhang L. et al., 2025). These challenges may profoundly affect their level of Job-Esteem and, consequently, their performance in preserving patient dignity. Therefore, this study focuses on Chinese nurses and proposes a core hypothesis: after controlling for the influence of Agreeableness, Job-Esteem among Chinese nurses is still independently and positively associated with their behaviors in maintaining patient dignity.

Maintaining patient dignity is a core nursing value and a moral responsibility of nurses. To systematically evaluate nurses‘ performance in this aspect, Yea-Pyng Lin et al. developed the Dignity in Care Scale for Nurses (Lin and Tsai, 2019). Through literature review and qualitative research, this scale identifies multiple key dimensions of Chinese nurses' behaviors for maintaining patient dignity (e.g., Communication Skills for Emotional Support, Confidentiality of Patient Information, Patient Care Needs Promptly, Respects for Patients Anatomy, Safe Environment for the Patient, and Protect the Patient's Wellbeing). However, existing research has primarily focused on validating the scale's reliability and validity, without in-depth exploration of the relative contribution of each behavioral dimension to patients‘ overall perception of dignity. Clarifying the differential contribution rates of each dimension in maintaining patient dignity holds significant theoretical and practical value. In situations where clinical resources are limited, identifying the behavioral dimensions with the highest impact weight on patient dignity can help nursing managers allocate resources more precisely and set training priorities, thereby making nurses' investments more cost-effective. For nursing educators, understanding the core contributing dimensions can provide an empirical basis for curriculum design and competency development. Therefore, to deepen the understanding of Nurses‘ behaviors for maintaining patient dignity and guide practical application, the second objective of this study is to move beyond mere dimension identification and quantitatively analyze the relative contribution strength of each behavioral dimension to patients' overall perception of dignity.

In summary, this study is committed to achieving two interrelated objectives: (1) to explore the relationship between Job-Esteem of nurses and their behaviors for maintaining patient dignity, so as to fill the key gap in understanding the driving forces behind this core care behavior; (2) to quantitatively determine the relative contribution of different dimensions of Nurses‘ behaviors for maintaining patient dignity to patients' overall perception of dignity. Going beyond mere dimension identification, this study provides a basis for setting priorities in practice. By integrating these two perspectives—exploring the intrinsic psychological antecedent (Job-Esteem) and evaluating the effectiveness of external behaviors (dimension contribution)—this study aims to gain a more comprehensive understanding of the intrinsic mechanisms of maintaining patient dignity in nursing practice.

## 2 Methods

### 2.1 Study design and participants

The first round of data collection for this study was conducted in April 2025, aiming to obtain preliminary data and exploratory findings for the research. A survey was conducted in the Second Affiliated Hospital of Bengbu Medical University (Anhui Province, China), a tertiary grade A general hospital with more than 500 beds that integrates medical treatment, teaching, and scientific research. Using the convenience sampling method, a total of 540 questionnaires were collected at this stage. After eliminating 32 invalid questionnaires, 508 valid questionnaires were ultimately obtained, with an effective recovery rate of 94%.

To verify the preliminary research results and enhance the representativeness of the sample as well as the external validity of the research conclusions, we conducted the second round of data collection in November 2025. This time, a multi-stage random sampling method is adopted. The specific process is as follows:

Phase One: Institutional sampling. Firstly, 13 tertiary grade A general hospitals with more than 500 beds were screened from East China and Central China, in line with the national hospital classification standards. These hospitals are regional medical hubs providing comprehensive healthcare services, undertaking the diagnosis and treatment of critical and complex diseases, as well as medical education and scientific research tasks. From these institutions, 5 hospitals were randomly selected as candidate institutions by using the simple random sampling method.

Phase Two: Population sampling. In the five designated hospitals, participants were recruited using the convenience sampling method, and uniform inclusion and exclusion criteria were strictly implemented. A total of 496 valid questionnaires were obtained in this round of data collection. Inclusion criteria: (1) Nurses who hold a nurse qualification certificate and are currently employed; (2) Nurses who gave informed consent and voluntarily participated in this study. The exclusion criteria include: (1) Retired nurses, trainee nurses, intern nurses, and other nurses who do not directly provide patient care; (2) Nurses suffering from severe mental illness or taking psychotropic drugs.

To determine the required sample size to achieve appropriate statistical power, an *a priori* power analysis was conducted using G^*^Power v.3.1.9, with the following parameters: effect size *f*^2^ = 0.15 (Zheng et al., 2011), α = 0.05, power = 0.90, and the maximum number of predictor variables is 14. The minimum sample size required is calculated to be 166. However, this calculated value is the theoretical lower limit under ideal random sampling. Given that this study employs a multi-stage sampling method, to ensure the robustness of the model, we have set a sample size target far above this threshold. The final total sample size of this study (*N* = 1004) fully met this requirement, providing a solid statistical basis for the research conclusion.

### 2.2 Measurements

Dignity in Care Scale for Nurses (DICSN): This scale, developed by Lin and Tsai (2019), was used to evaluate nurses' behaviors in maintaining patient dignity. It consists of 36 items across six dimensions: communication Skills for Emotional Support (CSES) (e.g., “Help patients manage their emotions, be ethical, and reduce the patient's sense of loneliness and isolation”), Confidentiality of Patient Information (CPI) (e.g., “Protect the patient's personal information during hospitalization”), Patient Care Needs Promptly (PCNP) (e.g., “Obtain informed consent immediately when psychological needs arise during care”), Respects for Patients Anatomy (RPA) (e.g., “Respectfully ask patients if there are any beliefs or customs associated with their cultural background that should be considered during their care”), Safe Environment for the Patient (SEP) (e.g., “Provide a dedicated conference room for patients and their families to discuss their illness without interruption”), and Protect the Patient's Wellbeing (PPWB) (e.g., “Understand and adhere to the needs and wishes of each patient and their family”). Each item is rated on a 5-point Likert scale ranging from “Never” (1) to “Always” (5), with higher scores indicating a higher frequency of dignity-preserving care. To test the applicability of this scale among the nurse population in Chinese mainland, we verified its psychometric characteristics.

The verification results were established in a separate validation study.

In that study, expert evaluation showed that the item-level content validity index (I-CVI) ranged from 0.870 to 1.000, and the scale-level content validity index (S-CVI/Ave) was 0.974, indicating good content validity (Souza et al., 2017). Exploratory factor analysis yielded a KMO value of 0.910, a significant Bartlett's test (*p* < 0.001), and six extracted factors accounting for 70.760% of the total variance, with factor loadings ranging from 0.599 to 0.925. Confirmatory factor analysis demonstrated good model fit (Souza et al., 2017): χ^2^/df = 2.027, RMSEA = 0.054, CFI = 0.938, TLI = 0.933, IFI = 0.939. The overall Cronbach's α coefficient was 0.923, indicating excellent internal consistency (Souza et al., 2017). In the present study sample, the DCSN also exhibited excellent reliability. The overall Cronbach's α was 0.975. The item-total correlations ranged from 0.644 to 0.809, all exceeding the recommended criterion of 0.30 (Wang et al., 2024), confirming the scale's strong internal consistency.

Job-Esteem Scale for Nurses (JES-HN): Originally developed by Choi and Jung (2020) for Korean nurses in 2020, this study used the Chinese version translated by Shi and Shen (2021) to assess the level of Job-Esteem among nurses in China. The scale contains 25 items across five dimensions (professional self-awareness, professional competence, social trust and respect, respect and recognition of the organization, and professional authority and future value). Responses are recorded on a 5-point Likert scale from “Strongly Disagree” (1) to “Strongly Agree” (5), with higher scores indicating stronger Job-Esteem. In this study, the Cronbach's α was 0.970, item-total correlations ranging from 0.626 to 0.839 (Wang et al., 2024).

*Chinese Big Five Personality Inventory Brief Version (CBF-PI-B):* Developed by Wang et al. (2011), this inventory includes five trait subscales (extraversion, neuroticism, conscientiousness, openness, and agreeableness). This study used the agreeableness subscale (8 items) to measure nurses' agreeableness trait (e.g., “Despite the existence of dark aspects in human society (such as war, evil, and fraud), I still believe that human nature is generally good”). Items are rated on a 6-point scale from 1 (strongly disagree) to 6 (strongly agree), with higher scores indicating stronger agreeableness. In this study, the Cronbach's α was 0.711, item-total correlations ranging from 0.473 to 0.741 (Wang et al., 2024).

### 2.3 Data analysis

The preliminary data processing and intergroup comparisons were primarily performed using SPSS 26.0. Specifically, descriptive statistics were conducted; given the skewed distribution of the data, continuous variables were described in quartiles, and categorical variables were expressed as frequencies and percentages. Non-parametric tests were used to analyze the differences in Nurses' Behaviors to Maintain Patient Dignity across different demographic variables.

Secondly, a correlation matrix was constructed using JASP 0.19.1 to analyze the correlations among Nurses' Behaviors to Maintain Patient Dignity, nurses' Job-Esteem, and Agreeableness.

Subsequently, taking the Nurses' Behaviors to Maintain Patient Dignity as the dependent variable, and the sociodemographic variables with statistically significant differences in non-parametric tests, Agreeableness, and Job-Esteem as independent variables, a stratified regression analysis was conducted. Before the regression analysis, we conducted tests for linearity, normality and homogeneity of variance. Based on the residual scatter plot, Q-Q plot and P-P plot, all assumptions were satisfied. To further verify the structural relationship among the variables, a structural equation model was constructed using Amos 24.0. Taking Job-Esteem and Nurses' Behaviors to Maintain Patient Dignity as latent variables, and incorporating Agreeableness and demographic variables with statistical significance as control variables into the model to test the fitting degree of the overall model.

Finally, to determine the relative importance of each dimension of maintaining patient dignity, principal component analysis (PCA) was used to calculate the weights (Ben Salem and Ben Abdelaziz, 2021). The data were tested for KMO and Bartlett's sphericity, and then principal component analysis was conducted. The number of principal components was determined based on the cumulative contribution rate obtained from the principal component analysis (a comprehensive index with a cumulative contribution rate greater than 60% is considered a principal component). As only one principal component was extracted in this study, the weights were obtained by normalizing the absolute values of the loadings. All statistical tests in this study were judged to be statistically significant at *P* < 0.05.

## 3 Results

### 3.1 Participant characteristics

The majority of participants in this study were female (90.0%), and more than half were between 30 and <41 years old (55.0%). Most participants were married (77.2%) and held a bachelor's degree (87.9%). Additionally, 41.1% had 11 to 20 years of clinical experience, 49.0% held the title of nurse supervisor, and 14.5% reported an average monthly salary above ¥8,000. Regarding self-rated health, 48.9% reported being in fair health. Non-parametric tests showed that all the general information of the nurses was significantly associated with Nurses' Behaviors to Maintain Patient Dignity (*P* < 0.05) (Table 1).

**Table 1:** The participants' characteristics.

**Variables**	**Categories**	***N*(%)**	**NBMPD score *M*(*P*_25_, P_75_)**	**Statistical quantity value**	***P***
**Gender**				34807.500^a^	<0.001
	Male	100 (10.0)	155.00 (141.00,174.00)	
	Female	904 (90.0)	170.00 (145.00,180.00)	
**Age (Year)**				48.499^b^	<0.001
	<30	268 (26.7)	153.00 (141.00,175.00)	
	30~ <41	552 (55.0)	171.00 (145.25,180.00)	
	≥41	184 (18.3)	173.00 (158.25,180.00)	
**Marital status**				25.992^b^	<0.001
	Single	207 (20.6)	157.00 (143.00,175.00)	
	Married	775 (77.2)	170.00 (146.00,180.00)	
	Others	22 (2.2)	178.00 (153.25,180.00)	
**Educational level**				17.832^b^	<0.001
	Specialty	81 (8.1)	158.00 (143.00,174.00)	
	Undergraduate	883 (87.9)	170.00 (145.00,180.00)	
	Master's degree or above	40 (4.0)	153.00 (140.50,170.50)	
**Years of work experience**				64.247^b^	<0.001
	<5	117 (11.7)	152.00 (139.50,171.00)	
	5~10	331 (33.0)	158.00 (143.00,179.00)	
	11~20	413 (41.1)	174.00 (149.00,180.00)	
	>20	143 (14.2)	174.00 (160.00,180.00)	
**Professional title**				25.728^b^	<0.001
	Nurse	76 (7.6)	152.50 (141.00,173.75)	
	Nurse practitioner	395 (39.3)	161.00 (143.00,180.00)	
	Nurse supervisor	492 (49.0)	172.00 (148.25,180.00)	
	Associate nurse practitioner and above	41 (4.1)	169.00 (161.00,176.00)	
**Average monthly income**				25.198^b^	<0.001
	<4000	128 (12.7)	156.50 (144.00,175.75)	
	4000~6000	450 (44.8)	164.00 (144.00,180.00)	
	6000~8000	280 (27.9)	173.50 (151.00,180.00)	
	>8000	146 (14.5)	172.00 (151.00,180.00)	
**self-rated health status**				18.046^b^	0.001
	Very poor	19 (1.9)	158.00 (137.00,176.00)	
	Poor	89 (8.9)	156.00 (143.50,178.00)	
	Fair	491 (48.9)	166.00 (144.00,180.00)	
	Good	269 (26.8)	171.00 (148.50,180.00)	
	Very good	136 (13.5)	175.00 (145.00,180.00)	

Nurses' Behaviors to Maintain Patient Dignity (NBMPD). a: U 值, b: H 值.

### 3.2 Descriptive statistics of all variables and reliability coefficients of subscales

The total score of Nurses' Behaviors to Maintain Patient Dignity was 168.00(145.00,180.00), and the total score of Job-Esteem for Nurses was 112.00(99.00,124.00). The scores of each dimension are shown in Table 2. As shown in Table 2, nurses' ability to maintain patients' dignity is at a medium to high level, and their Job-Esteem is at a medium level. In addition, the reliability coefficients of each subscale are also presented in Table 2.

**Table 2:** Descriptive statistics for nurses' behaviors to maintain patient dignity, job-esteem, and agreeableness (*M*[*P*_25_, *P*_75_]).

**Variables**	**Number of items**	**Dimension score**	**Average score of items**	**Cronbach's Alpha**
1 NBMPD	36	168.00(145.00,180.00)	4.67(4.03,5.00)	0.975
CSES	11	52.00(44.00,55.00)	4.73(4.00,5.00)	0.966
CPI	6	30.00(25.00,30.00)	5.00(4.17,5.00)	0.882
PCNP	6	29.00(24.00,30.00)	4.83(4.00,5.00)	0.892
RPA	5	23.00(19.00,25.00)	4.60(3.80,5.00)	0.841
SEP	3	14.00(12.00,15.00)	4.67(4.00,5.00)	0.827
PPWB	5	25.00(20.00,25.00)	5.00(4.00,5.00)	0.899
2 Job-Esteem	25	112.00(99.00,124.00)	4.48(3.96,4.96)	0.970
Procompetence	10	46.00(40.00,50.00)	4.60(4.00,5.00)	0.947
Proselfaware	4	19.00(16.00,20.00)	4.75(4.00,5.00)	0.924
Soctrustrespect	3	15.00(12.00,15.00)	5.00(4.00,5.00)	0.901
Orgrespectrecog	4	17.00(15.00,20.00)	4.25(3.75,5.00)	0.941
Proauthfutureval	4	16.00(13.00,20.00)	4.00(3.25,5.00)	0.928
3 Agreeableness	8	36.00(32.00,42.00)	4.50(4.00,5.25)	0.711

Nurses' Behaviors to Maintain Patient Dignity (NBMPD), Communication Skills for Emotional Support (CSES), Confidentiality of Patient Information (CPI), Patient Care Needs Promptly (PCNP), Respects for Patients Anatomy (RPA), Safe Environment for the Patient (SEP), and Protect the Patient's Well-Being (PPWB). professional competence (ProCompetence), professional self-awareness (ProSelfAware), social trust and respect (SocTrustRespect), respect and recognition of the organization (OrgRespectRecog), and professional authority and future value (ProAuthFutureVal).

### 3.3 Correlation analysis

**Figure 1:**
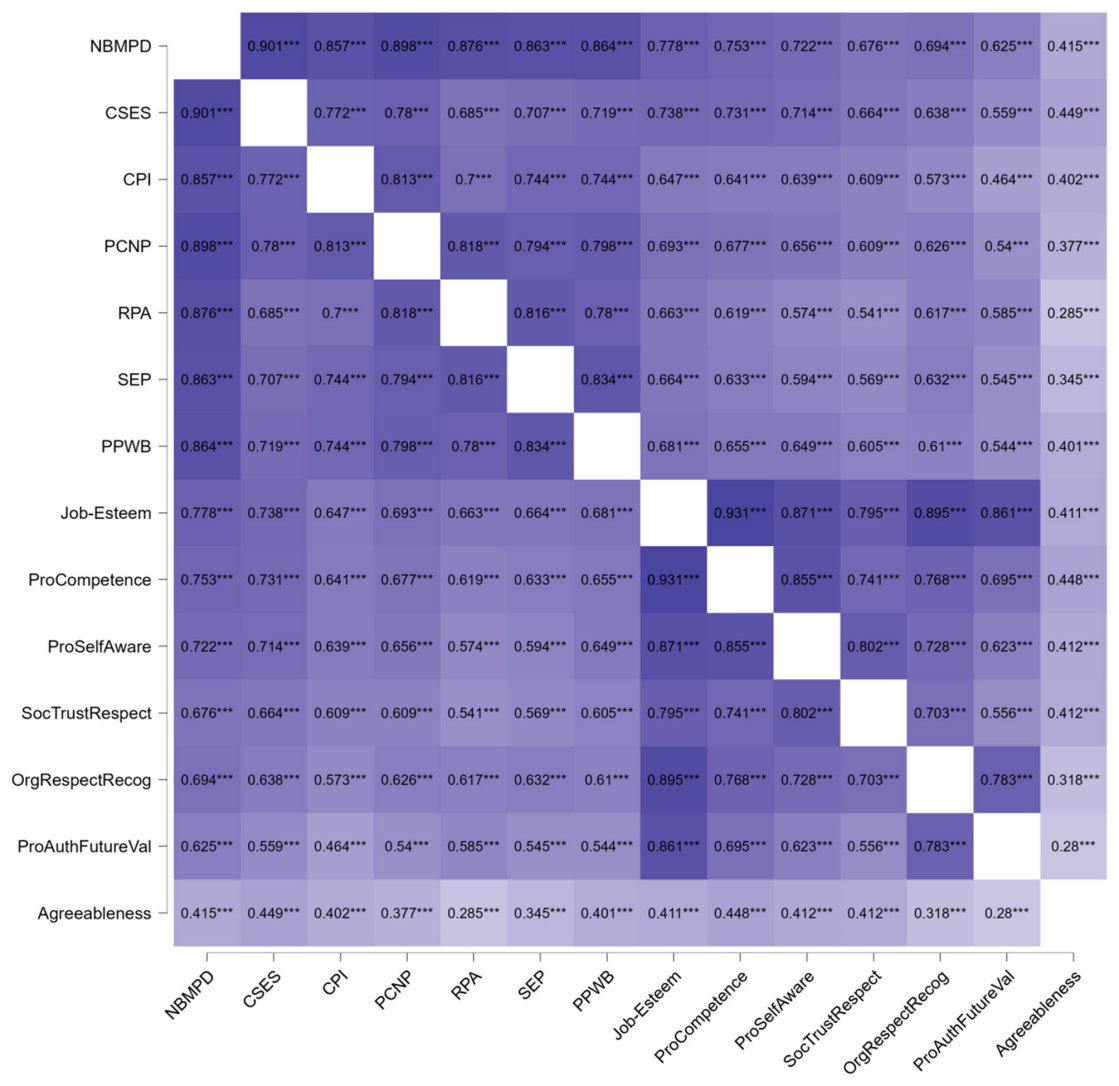
The correlation matrix between job-esteem, agreeableness and nurses' behaviors to maintain patient dignity. The matrix is visualized using a purple gradient heat map. Color depth: indicates the strength of the correlation. The darker the color, the stronger the positive correlation. The lighter the color, the weaker the correlation. Value: the specific value in the cell is the Spearman correlation coefficient. All coefficients in this study are positive, indicating a positive correlation among the variables. ^***^p < 0.001. All variable abbreviations used in this figure can be found in the abbreviation table provided in the Supplementary material.

As shown in Figure 1, Nurses‘ Behaviors to Maintain Patient Dignity and dimensions are significantly positively correlated with Job-Esteem for Nurses and dimensions, as well as Agreeableness (*P* < 0.001). Compared with Agreeableness, the correlation between Job-Esteem and the behavior of maintaining patients' dignity is stronger (*r* = 0.778, *P* < 0.001).

### 3.4 Hierarchical regression analysis

This study employed hierarchical regression analysis, aimed to examine the independent predictive effect of nurses‘ Job-Esteem on Nurses' Behaviors to Maintain Patient Dignity after controlling for demographic variables and the personality trait of Agreeableness. First, the sociodemographic variables that showed statistically significant differences in Nurses‘ Behaviors to Maintain Patient Dignity in nonparametric tests were entered into the first layer of the hierarchical regression analysis. Next, Agreeableness was input into the second layer; finally, the five dimensions of nurses' Job-Esteem were incorporated into the third layer. All variance inflation factor (VIF) values were between 1 and below the critical threshold of 10, indicating no multicollinearity. The results of the hierarchical regression are presented in Table 3.

**Table 3:** Hierarchical regression analysis.

**Model**	**Variables**	**B**	**SE**	**β**	***R^2^***	**Adjust *R^2^***	***ΔR^2^***	***p***	**95%*CI***
1	(Constant)	118.99	5.77		0.10	0.09	0.10	0.00	(107.66,130.31)
	Gender	5.82	2.25	0.08				0.01	(1.41,10.23)
	Age (Year)	3.00	1.67	0.10				0.07	(−0.29,6.28)
	Married	0.70	1.85	0.01				0.71	(−2.94,4.33)
	Others	6.24	4.53	0.04				0.17	(−2.64,15.13)
	Undergraduate	3.68	2.40	0.06				0.13	(−1.03,8.39)
	Master's degree or above	−2.72	4.00	−0.03				0.50	(−10.57,5.12)
	Years of work experience	3.31	1.38	0.14				0.02	(0.60,6.01)
	Nurse practitioner	−1.46	2.70	−0.03				0.59	(−6.76,3.84)
	Nurse supervisor	−3.29	3.17	−0.08				0.30	(−9.50,2.93)
	Associate nurse practitioner and above	−7.82	4.81	−0.08				0.10	(−17.27,1.62)
	Average monthly income	2.02	0.79	0.09				0.01	(0.47,3.58)
	self-rated health status	3.00	0.71	0.13				0.00	(1.61,4.38)
2	(Constant)	93.06	5.76		0.22	0.21	0.12	0.00	(81.76,104.36)
	Gender	2.94	2.10	0.04				0.16	(−1.19,7.07)
	Age (Year)	1.61	1.56	0.05				0.30	(−1.46,4.67)
	Married	0.11	1.72	0.00				0.95	(−3.28,3.49)
	Others	4.95	4.21	0.04				0.24	(−3.32,13.21)
	Undergraduate	3.22	2.23	0.05				0.15	(−1.16,7.60)
	Master's degree or above	−2.25	3.72	−0.02				0.54	(−9.55,5.04)
	Years of work experience	2.56	1.28	0.11				0.05	(0.04,5.08)
	Nurse practitioner	−1.93	2.51	−0.05				0.44	(−6.86,3.00)
	Nurse supervisor	−3.38	2.95	−0.08				0.25	(−9.16,2.41)
	Associate nurse practitioner and above	−7.48	4.48	−0.07				0.10	(−16.27,1.31)
	Average monthly income	1.10	0.74	0.05				0.14	(−0.36,2.56)
	self-rated health status	1.80	0.66	0.08				0.01	(0.50,3.10)
	Agreeableness	1.18	0.09	0.37				0.00	(0.99,1.37)
3	(Constant)	25.59	4.51		0.64	0.64	0.42	0.00	[(6.73,34.45)
	Gender	4.89	1.43	0.07				0.00	(2.07,7.70)
	Age (Year)	0.37	1.06	0.01				0.73	(−1.71,2.46)
	Married	−0.64	1.17	−0.01				0.59	(−2.94,1.66)
	Others	1.50	2.87	0.01				0.60	(−4.14,7.13)
	Undergraduate	−0.14	1.52	0.00				0.93	(−3.12,2.84)
	Master's degree or above	−4.46	2.54	−0.04				0.08	(−9.45,0.54)
	Years of work experience	1.01	0.88	0.04				0.25	(−0.71,2.73)
	Nurse practitioner	−4.81	1.72	−0.11				0.01	(−8.19, −1.44)
	Nurse supervisor	−6.25	2.02	−0.15				0.00	(−10.20, −2.29)
	Associate nurse practitioner and above	−5.65	3.07	−0.05				0.07	(−11.68,0.38)
	Average monthly income	0.78	0.51	0.03				0.13	(−0.22,1.78)
	self-rated health status	−0.46	0.46	−0.02				0.32	(−1.37,0.44)
	Agreeableness	0.26	0.07	0.08				0.00	(0.13,0.40)
	Procompetence	1.04	0.16	0.29				0.00	(0.74,1.35)
	Proselfaware	1.54	0.38	0.18				0.00	(0.78,2.29)
	Soctrustrespect	1.62	0.39	0.15				0.00	(0.87,2.38)
	Orgrespectrecog	1.22	0.220	0.19				0.00	(0.79,1.65)
	Proauthfutureval	0.11	0.17	0.02				0.512	(−0.22,0.44)

B, Unstandardized Regression Coefficient; SE, Standard Error; β, Standardized Regression Coefficient. All variable abbreviations used in this figure can be found in the abbreviation table provided in the Supplementary material.

In Model 1, only sociodemographic variables were included, and the model was significant: R2 = 0.10 (adjusted R^2^ = 0.09), F = 9.027, *P* < 0.001. In the second step of the regression analysis, after incorporating Agreeableness, the model was significant: R^2^ = 0.22 (adjusted R^2^ = 0.21), ΔR^2^ = 0.12, F = 21.568, *P* < 0.001. The final model indicates that even after controlling for sociodemographic variables and Agreeableness, Job-Esteem for Nurses is still significantly correlated with Nurses‘ Behaviors to Maintain Patient Dignity: R^2^ = 0.64 (adjusted R^2^ = 0.64), ΔR^2^ = 0.42, F = 97.966, *P* < 0.001. This indicates that the dimensions of Job-Esteem explained an additional 42% of the variance in Nurses' Behaviors to Maintain Patient Dignity, above and beyond the predictors in Model 2.

Furthermore, after controlling for demographic variables and Agreeableness, all dimensions of Job-Esteem, except for professional authority and future value, are significant positive predictors of Nurses' Behaviors to Maintain Patient Dignity.

### 3.5 Structural equation modeling analysis

The Structural Equation Model demonstrated a good fit to the data [χ^2^/df = 3.838 (Sathyanarayana and Mohanasundaram, 2024), CFI = 0.958, TLI = 0.944, RMSEA = 0.075 (Wen et al., 2004)]. The standardized path coefficient from the Job-Esteem latent variable to the Dignity Behaviors latent variable was statistically significant (β = 0.767, *p* < 0.001).This result robustly supports our core hypothesis that nurses' Job-Esteem is a significant positive predictor of their behaviors in maintaining Patient dignity, even after controlling for the effects of demographic variables and Agreeableness within the SEM framework. The path diagram of the structural equation is shown in Supplementary Figure 1.

### 3.6 Total variance explained

The Kaiser–Meyer–Olkin (KMO) measure was 0.909, and Bartlett's test of sphericity was significant (*P* < 0.001), indicating that the data were suitable for principal component analysis (PCA). The initial eigenvalues, percentage of variance, and cumulative variance explained are presented in Table 4. One principal component with an eigenvalue greater than 1.0 was extracted, accounting for 79.236% of the total variance.

**Table 4:** Total variance explained by nurses' behaviors to maintain patient dignity.

**Component**	**Initial eigenvalues**			**Extraction sums of squared loadings**		
	**Total**	**% of Variance**	**Cumulative %**	**Total**	**% of Variance**	**Cumulative %**
1	4.754	79.236	79.236	4.754	79.236	79.236
2	0.461	7.682	86.918			
3	0.244	4.060	90.978			
4	0.236	3.931	94.909			
5	0.169	2.812	97.720			
6	0.137	2.280	100.000			

### 3.7 Weight calculation

Principal component loadings represent the correlation between each dimension and the principal component. Weights were obtained by normalizing the absolute values of the loadings, using the formula:$W_{i}=\frac{\left|Loading_{i}\right|}{\overset{6}{\underset{j=1}{\sum }}|Loading_{j}|}$, where *W*_*i*_ is the weight of the i-th dimension. The results are shown in Table 5. The weight range of the dimensions was 16.04% to 17.50%, among which “Patient Care Needs Promptly (PCNP)” had the highest weight (17.50%), and “Communication Skills for Emotional Support (CSES)” had the lowest weight (16.04%).

**Table 5:** Weight results of each dimension.

**Dimension**	**loadings**	**Weight(%)**
CSES	0.856	16.04%
CPI	0.875	16.39%
PCNP	0.934	17.50%
RPA	0.868	16.26%
SEP	0.899	16.84%
PPWB	0.906	16.97%

All variable abbreviations used in this figure can be found in the abbreviation table provided in the Supplementary material.

## 4 Discussion

This study first delineates the current state and shortfalls in Chinese Nurses' Behaviors to Maintain Patient Dignity. Subsequently, building upon the control of key variables, it provides an in-depth analysis of the unique predictive effects and potential mechanisms of various dimensions of Job-Esteem on these dignity-preserving behaviors. Finally, the relative importance of each dimension within dignity-preserving behaviors was determined through weight analysis.

First, the total score of Chinese Nurses' Behaviors to Maintain Patient Dignity falls at a moderate to high level, yet there remains room for improvement. This study found that among the various dimensions of Nurses' Behaviors to Maintain Patient Dignity, the dimension of “Communication Skills for Emotional Support (CSES)” scored the highest. In contrast, the dimension score for “Safe Environment for the Patient (SEP)” is the lowest. A qualitative study by Lin and Tsai (Asmaningrum and Tsai, 2018) also noted that providing emotional support is a means of maintaining patient dignity. Using a humorous communication style to offer patients words of encouragement and comfort not only helps patients feel respected but also promotes their recovery (Asmaningrum and Tsai, 2018). Our findings reveal that dignity maintenance in medical services is primarily reflected in nursing care, with relatively less attention paid to the environmental aspects that enhance the sense of dignity. A possible reason for this is the significant pressure in China's medical environment, characterized by an imbalanced nurse-to-patient ratio and excessive workloads for nurses, which often makes it difficult for them to attend to the maintenance of environmental details (Zhu et al., 2022). Most patients and their family members have reported that a satisfactory medical environment and facilities are key factors in behaviors that maintain patient dignity, while unsanitary and noisy environments make them feel their dignity has been violated (Tehranineshat et al., 2020). The physical environment serves as a crucial carrier for the experience of dignity. To improve the overall level of Chinese Nurses' Behaviors to Maintain Patient Dignity, it is urgent to address the deficiency in environmental safety maintenance.

The research results indicate that demographic factors such as nurses' gender, years of work experience, professional titles, average monthly income, and self-rated health status have an impact on their behaviors for maintaining patient dignity. This is consistent with the findings of previous studies (Jiang et al., 2015; Norful et al., 2024; Zhang X. et al., 2025). The data analysis of this study shows that there is a high correlation between years of work experience and their professional titles. Given this strong correlation, and since previous literature (Teng et al., 2024) clearly states that nurses' humanistic care competence improves with the increase in years of work experience, we believe that years of work experience comprehensively reflects changes in experience accumulation and professional maturity, and is a core variable influencing behaviors for maintaining patient dignity. This conclusion is supported by the study conducted by Zhu et al. (2022), which shows that for Chinese nursing students, in-depth clinical experience can significantly enhance their understanding of humanistic care as the core of high-quality nursing.In addition, this study also finds that nurses' self-rated health status is an important influencing factor of their behaviors for maintaining patient dignity. This highly echoes the findings of a study on Japanese nurses (Arakawa et al., 2011), which indicates that nurses' physical and mental health status directly affects their work performance, as well as patient safety and nursing quality. Therefore, improving nurses' physical and mental health status is an important foundation for ensuring their effective behaviors in maintaining patient dignity.

Beyond individual characteristics, the core of this study lies in exploring the connection between Nurses' Behaviors to Maintain Patient Dignity and their intrinsic job feelings in China. This study focuses on the Chinese nurse population and, under the control of demographic variables and Agreeableness, verifies the association between Nurses' Behaviors to Maintain Patient Dignity and Job-Esteem of nurses. The research found that after controlling for demographic variables and Agreeableness, the addition of Job-Esteem additionally explained 42% of the total variation in Nurses' Behaviors to Maintain Patient Dignity. This result suggests that Job-Esteem may play a core role in Nurses' Behaviors to Maintain Patient Dignity. This finding is consistent with previous studies on the relationship between nurses' prosocial behaviors and their own dignity perception (Haddock, 1996; You, 2017). Among the five dimensions of Job-Esteem, professional competence is the strongest predictor of Chinese Nurses' Behaviors to Maintain Patient Dignity. Professional competence, as a comprehensive reflection of nurses' skills, knowledge, attitudes, values, and abilities (Zaitoun, 2024), serves as the core driver for upholding patients' dignity. Nurses with a strong sense of professional competence are able to deliver high-quality care, effectively safeguard patient safety, and contribute to positive healthcare outcomes (Zaitoun, 2024). Empathic care, for instance, is a typical manifestation of such comprehensive competence. It enables nurses to forge genuine connections with patients, leveraging spiritual care to alleviate patients' stress and anxiety while upholding their dignity (Babaii et al., 2021). More importantly, this sense of competence empowers nurses to apply their knowledge and skills with greater confidence and efficiency when addressing clinical challenges, thereby further enhancing their self-efficacy (Tong et al., 2024) and ultimately translating into improved patient care outcomes.

Secondly, respect and recognition of the organization (referring to the nurse's evaluation of their profession's status, role, and level of recognition within the organization (Shi and Shen, 2021), professional self-awareness, and social trust and respect were also significant positive predictors. At the organizational level, the experience of respect from the work environment is crucial—for instance, recognition from management, fair treatment, participation in decision-making, and resource support. Organizational Support Theory (OST) posits that when employees perceive that the organization values their contributions and cares about their wellbeing, they develop a sense of obligation to reciprocate with positive attitudes and behaviors (Rhoades and Eisenberger, 2002). Specifically, nurses perceiving organizational value and support significantly enhances their energy, confidence, and sense of security, thereby enabling them to focus more effectively on patient needs and dignity. Multiple studies confirm that perceived organizational support and organizational commitment among nurses improve their work attitudes, enhance self-efficacy and work engagement, and ultimately elevate the quality of care (Al-Hamdan and Issa, 2022; Tang et al., 2022). Therefore, for medical institutions/organizations, it is recommended to establish a regular recognition and incentive mechanism, grant nurses the right to participate in decision-making, and provide sufficient resources and psychological support.

Professional self-awareness embodies the significance of nurses pursuing self-worth in nursing practice (Shi and Shen, 2021). According to self-determination theory, when basic psychological needs are met in a supportive social environment, it will stimulate vitality, self-motivation, and happiness (Ryan, 2009). When nurses highly identify with their professional identity and value, they are more likely to view maintaining patient dignity as a core intrinsic requirement of their role rather than an external task. The results of this study are consistent with those of Taskiran Eskici et al. (2025): the enhancement of the importance of nurses' professional values not only improves moral sensitivity and the perception of nursing quality but, more importantly, transforms ethical requirements such as maintaining dignity into an intrinsic component of professional identity, making caring behaviors more spontaneous and stable. At the social level, societal trust and respect are equally crucial. Workplace incivility—such as disrespectful behaviors from patients and their family members—stands out as a prominent manifestation of societal distrust and disrespect toward nurses (Alquwez, 2020), and such negative experiences significantly undermine the foundation for nurses to practice behaviors that maintain patient dignity. A socially supportive environment is an essential guarantee for nurses to sustain their engagement in caring behaviors. Studies have shown that nurses who experience incivility exhibit reduced job satisfaction, increased burnout, and diminished compassionate care; this, in turn, impairs nursing quality and even endangers patient safety (Kavakli and Yildirim, 2022; Lee et al., 2024).

Interestingly, the dimension of professional authority and future value did not demonstrate a significant predictive effect on Nurses' Behaviors to Maintain Patient Dignity. This outcome underscores the complexity of how Job-Esteem of nurses influences nursing conduct and highlights a crucial barrier in translating macro-level professional perceptions into micro-level ethical actions—namely, the moral dilemmas frequently encountered by nurses in clinical practice. Although the nursing profession is often accorded lofty social accolades such as “Angels in White,” individual nurses often occupy a relatively vulnerable position within the clinical decision-making hierarchy, and their professional worth is frequently undervalued (Lee et al., 2023). At the same time, when confronted with the stark realities of high-intensity workloads, staffing shortages, and ethical conflicts, the motivating power of believing in a bright professional future may become abstract or seem unattainable. These factors collectively contribute to a profound moral dilemma: even when nurses deeply acknowledge the macro-level value and authority of their profession, they often feel powerless in specific situations to fully fulfill the ethical demands of preserving patient dignity. Maintaining patient dignity highly depends on nurses' ethical sensitivity and their exercise of autonomy during immediate patient interactions (Jang et al., 2022). However, practical moral challenges—such as balancing routine care with dignity preservation, managing excessive workloads, and navigating complex interprofessional relationships—can significantly deplete nurses' psychological resources (Haahr et al., 2020), exacerbate burnout, and constrain their scope for professional autonomy. Within such constraints, macro-level perceptions of professional authority and future value struggle to directly translate into the intrinsic motivation needed to overcome immediate obstacles and drive concrete ethical decision-making and behavior.

Furthermore, this study quantified the relative importance of each dimension within Nurses‘ Behaviors to Maintain Patient Dignity through weighting analysis. The results indicated that the Patient Care Needs Promptly (PCNP) dimension carried the highest weight, suggesting it contributes most significantly to preserving patient dignity. This finding aligns with previous research, which has shown that timely responsiveness to patients' care needs is closely associated with their in-hospital dignity experience (Asmaningrum and Tsai, 2018). Patients widely perceive that prompt response to and fulfillment of their needs by nursing staff is a crucial component of receiving dignified care (Asmaningrum and Tsai, 2018). Multiple studies advocate for implementing “person-centered care,” positing that it effectively enhances patients‘ sense of respect (Asmaningrum and Tsai, 2018; Corazzini et al., 2019). Therefore, nursing personnel must regard prioritizing and meeting patients' reasonable needs as a core professional responsibility. This does not imply, however, that other dimensions (such as protecting privacy and respecting autonomy) can be overlooked in upholding patient dignity. Thus, while ensuring fundamental care needs are prioritized, nursing staff must also comprehensively attend to and appropriately manage all relevant dimensions. Only through such a holistic approach can the dignity experience of inpatients be genuinely enhanced, ultimately fostering a positive nurse-patient relationship.

## 5 Implications

The results of this study indicate a close association between nurses‘ Job-Esteem and Nurses' Behaviors to Maintain Patient Dignity. Professional competence exhibits the strongest predictive power among Job-Esteem's five dimensions. Organizational respect and recognition, professional self-awareness, and social trust and respect also emerge as significant positive predictors of this behavior. Based on the above findings and in combination with the design characteristics of cross-sectional studies, this study offers practical implications from the following aspects. All suggestions are based on the correlations among variables and do not involve causal inferences.

Healthcare institutions can optimize the clinical environment for safety and functionality while also integrating environmental supports that align with nurses' professional development. Specific measures include ensuring the integrity of examination curtains to protect patient privacy, conducting regular inspection and maintenance of medical equipment to minimize operational risks, promoting “Quiet Ward” initiatives to enhance the healing environment, and standardizing cleaning protocols to improve overall care quality. Existing research suggests that such physical environment improvements not only help safeguard patient dignity but may also create more favorable conditions for nurses to fully utilize their professional competencies (Ferri et al., 2015).

Nursing managers should focus on strengthening the professional competency building and health support systems for early-career nurses. It is advisable to systematically integrate education on dignity ethics, clinical mentoring by senior nurses (Smith et al., 2009), and reflective training based on typical case studies to enhance nurses‘ ethical sensitivity and clinical judgment. Concurrently, institutions should establish routine health support mechanisms, such as providing psychological counseling resources, stress management training, and flexible scheduling, thereby fostering a positive correlation with the sustenance of nurses' professional capabilities and their sense of professional identity.

Hospital management can design multi-dimensional intervention strategies centered on fostering a sense of professional respect, particularly focusing on dimensions such as professional competence and organizational recognition. Practical initiatives may include organizing ethics seminars, implementing outstanding nurse recognition awards, encouraging reflective practice, and appropriately involving nurses in the process of drafting relevant protocols to strengthen their professional identity. Furthermore, providing specialized training in communication skills, cross-cultural nursing, and ethical decision-making can bolster nurses' confidence in upholding patient dignity in complex clinical situations.

Healthcare institutions could consider collaborating with media partners to actively promote the image and value of the nursing profession, thereby establishing a virtuous cycle with societal trust and respect. When conditions permit, health administrative departments might also explore the gradual incorporation of professional respect-related metrics into nursing quality evaluations and vocational education systems, thereby guiding policy and resource allocation appropriately toward these critical areas.

By systematically integrating professional competency building, organizational empowerment, health support, and social image shaping, healthcare institutions may further stimulate nurses' proactive inclinations in safeguarding patient dignity, thereby holistically advancing the quality of humanistic care in clinical practice.

## 6 Limitations

This study has several limitations. First, the cross-sectional design limits a deeper exploration of the dynamic evolution and temporal relationship between Nurses' Behaviors to Maintain Patient Dignity and their Job-Esteem. Future research employing longitudinal designs will help uncover the temporal sequences and dynamic correlations between these variables, thereby providing insights with stronger predictive relevance for practical application. Second, Despite the multi-stage sampling and inclusion of tertiary grade A general hospitals, this study has sample-related limitations. Samples were only from Central and East China (excluding North, Northwest, Southwest, Northeast China)—regional disparities in healthcare, economy, and health awareness may limit national generalizability of conclusions. Future research should expand geographic coverage, include diverse-level medical institutions and cross-cultural comparisons to enhance representativeness and external validity. Finally, there are certain limitations in the control of variables. Although key demographic variables and the personality trait of Agreeableness were controlled, several important organizational and contextual factors, such as department type, shift schedule, nurse-to-patient ratios, specific manifestations of management support, and nurses' experiences of workplace incivility, were not included as covariates. These factors may not only influence nurses' levels of Job-Esteem but also affect Nurses' Behaviors to Maintain Patient Dignity, representing potential confounding effects. If future studies can systematically collect and control for these contextual variables, they will be able to more accurately reveal the independent impact of Job-Esteem on Nurses' Behaviors to Maintain Patient Dignity and to deeply explore the mechanisms through which the organizational environment operates in this process.

## 7 Conclusions

This study provides, for the first time, empirical evidence on the association between Job-Esteem and Nurses' Behaviors to Maintain Patient Dignity among nurses in tertiary grade A general hospitals in Central and East China. The results show that sociodemographic variables such as nurses' gender, work experience, professional title, average monthly income, and self-rated health status are all significantly associated with Nurses' Behaviors to Maintain Patient Dignity. Overall, there is still room for continuous improvement in the performance of nurses in tertiary grade A general hospitals in Central and East China in maintaining patient dignity.

After controlling for key covariates, multiple dimensions of Job-Esteem have been shown to be correlated with dignity-preserving nursing behaviors among nurses in the aforementioned tertiary grade A general hospitals in Central and East China. Among these, professional competence exhibits the strongest correlation, suggesting that professional capabilities may play a prominent role in ethical practice for this group. Furthermore, three dimensions—professional self-awareness, organizational respect and recognition, and social trust and respect—are all significantly positively correlated with dignity-preserving nursing behaviors. This indicates that nurses' internal professional cognition, the organizational climate of recognition, and social trust and respect together constitute important factors associated with ethical behaviors among nurses in these institutions.

Notably, no significant association was found between professional authority, future value, and dignity-preserving nursing behaviors in this study among the sampled nurses from tertiary grade A general hospitals in Central and East China. Further analysis revealed that Patient Care Needs Promptly (PCNP) remains the most influential dimension in maintaining patient dignity for the nurses in this study. However, to achieve systematic and sustainable dignity-preserving nursing practices among nurses in tertiary grade A general hospitals in Central and East China, joint efforts are still needed in multiple aspects, including professional competence development, organizational culture shaping, and social trust building.

## Data Availability

The original contributions presented in the study are included in the article/Supplementary material, further inquiries can be directed to the corresponding author/s.
