# Correction: Weight analysis of Chinese nurses' behaviors to maintain patient dignity and its relationship with job-esteem: a cross-sectional study controlling for agreeableness

**DOI:** 10.3389/fpsyg.2026.1785199

**Published:** 2026-01-28

**Authors:** Cong Guo, Chunlin Zhang, Cuizhu Zhou, Mengqi Zhu, Lingling Chen, Youran Liu, Yequn Zhang, Jie Wang, Tengfei Liang

**Affiliations:** 1 School of Nursing, Bengbu Medical University, Anhui, Bengbu, China; 2 The Second Affiliated Hospital of Bengbu Medical University, Anhui, Bengbu, China; 3 The Nethersole School of Nursing, Faculty of Medicine, The Chinese University of Hong Kong, Hong Kong SAR, China

**Keywords:** agreeableness, cross-sectional study, job-esteem, nurses, patient dignity

The files “Supplementary Table 1,” “Supplementary Table 2,” “Supplementary Table 3,” “Supplementary Table 4,” and “Supplementary Table 5” were erroneously published with the original version of this paper. These files have now been replaced with “Supplementary File 1.”

The original version of this article has been updated.

