# Supplementary material for: Weight analysis of Chinese nurses' behaviors to maintain patient dignity and its relationship with job-esteem: a cross-sectional study controlling for agreeableness
Source: Front Psychol. 2026 Jan 7;16:1710563. doi: 10.3389/fpsyg.2025.1710563 (PMC12819281; doi:10.3389/fpsyg.2025.1710563)
Supplement: Supplementary file 1 [file Supplementary_file_1.docx]

Supplementary Material

# Supplementary Figures and Tables

## Supplementary Figures


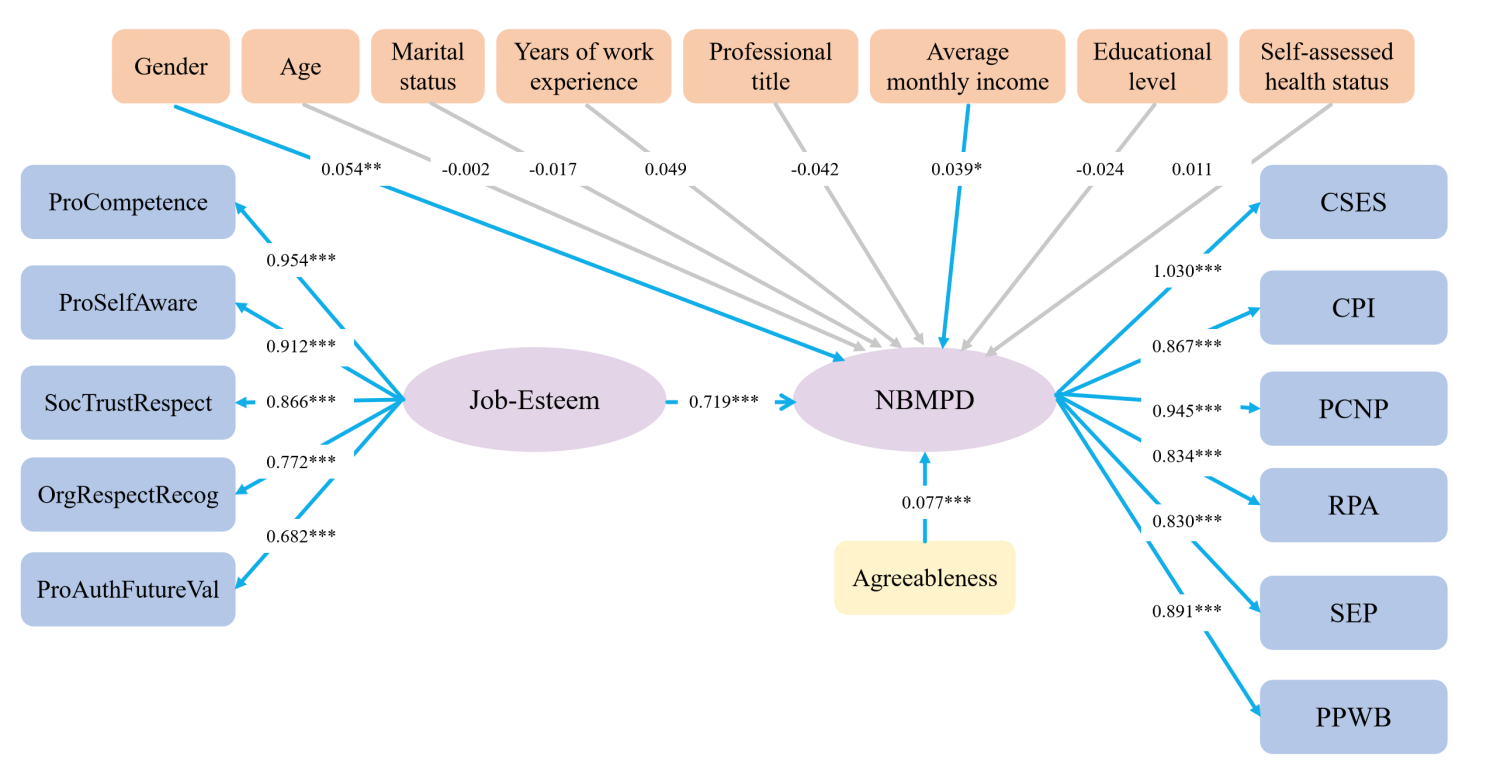


**Supplementary Figure 1.** Structural equation model path diagram. Blue lines indicate that the path is significant, while gray lines indicate that the path is not. ^*^*p* < 0.05, ^**^*p* < 0.01, ^***^*p* < 0.001. Nurses' Behaviors to Maintain Patient Dignity (NBMPD), Communication Skills for Emotional Support (CSES), Confidentiality of Patient Information (CPI), Patient Care Needs Promptly (PCNP), Respects for Patients Anatomy (RPA), Safe Environment for the Patient (SEP), and Protect the Patient’s Well-Being (PPWB). professional competence (ProCompetence), professional Self-awareness (ProSelfAware), social trust and respect (SocTrustRespect), respect and recognition of the organization (OrgRespectRecog), and professional authority and future value (ProAuthFutureVal).

## Supplementary Tables

**Table S1** Correlation coefficient between variables and Confidence Interval

| **Variables** |  | **1** | **2** | **3** |
| --- | --- | --- | --- | --- |
| 1 NBMPD | Spearman correlation | 1 |  |  |
|  | 95%CI | - |  |  |
| 2 Job-Esteem | Spearman correlation | 0.778*** | 1 |  |
|  | 95%CI | [0.747,0.805] | - |  |
| 3 Agreeableness | Spearman correlation | 0.415*** | 0.411*** | 1 |
|  | 95%CI | [0.356,0.468] | [0.355,0.467] | - |

Note: ****P*<0.001. Confidence Interval (CI) are reported at 95%. Nurses' Behaviors to Maintain Patient Dignity (NBMPD).

**Table S2** Measurement Scales, Variables, Dimensions, and Abbreviations in the Study

| **Scale Name** | **Measurement variable** | **Dimensions** |
| --- | --- | --- |
| Dignity in Care Scale for Nurses (DICSN) | Nurses' Behaviors to Maintain Patient Dignity (NBMPD) | Communication Skills for Emotional Support (CSES) |
|  |  | Confidentiality of Patient Information (CPI) |
|  |  | Patient Care Needs Promptly (PCNP) |
|  |  | Respects for Patients Anatomy (RPA) |
|  |  | Safe Environment for the Patient (SEP) |
|  |  | Protect the Patient’s Well-Being (PPWB) |
| Job-Esteem Scale for Nurses (JES-HN) | Job-Esteem | Professional self-awareness (ProSelfAware) |
|  |  | Professional competence (ProCompetence) |
|  |  | Social trust and respect (SocTrustRespect) |
|  |  | Respect and recognition of the organization (OrgRespectRecog) |
|  |  | professional authority  and future value (ProAuthFutureVal) |
| Chinese Big Five Personality  Inventory brief version (CBF-PI-B) | Agreeableness | Agreeableness Subscale |
